# Maintenance of Sorafenib following combined therapy of three-dimensional conformal radiation therapy/intensity-modulated radiation therapy and transcatheter arterial chemoembolization in patients with locally advanced hepatocellular carcinoma: a phase I/II study

**DOI:** 10.1186/1748-717X-5-12

**Published:** 2010-02-12

**Authors:** Jian-Dong Zhao, Jin Liu, Zhi-Gang Ren, Ke Gu, Zhen-Hua Zhou, Wen-Tao Li, Zhen Chen, Zhi-Yong Xu, Lu-Ming Liu, Guo-Liang Jiang

**Affiliations:** 1 Department of Radiation Oncology, Fudan University Shanghai Cancer Center, Shanghai, China; 2 Department of Integrative Oncology, Fudan University Shanghai Cancer Center, Shanghai, China; 3 Department of Interventional Radiology, Fudan University Shanghai Cancer Center, Shanghai, China

## Abstract

**Background:**

Three-dimensional conformal radiation therapy (3DCRT)/intensity-modulated radiation therapy (IMRT) combined with or without transcatheter arterial chemoembolization (TACE) for locally advanced hepatocellular carcinoma (HCC) has shown favorable outcomes in local control and survival of locally advanced HCC. However, intra-hepatic spreading and metastasis are still the predominant treatment failure patterns. Sorafenib is a multikinase inhibitor with effects against tumor proliferation and angiogenesis. Maintenance Sorafenib would probably prevent or delay the intrahepatic and extrahepatic spread of HCC after radiotherapy, which provides the rationale for the combination of these treatment modalities.

**Methods and design:**

Patients with solitary lesion (bigger than 5 cm in diameter) histologically or cytologically confirmed HCC receive TACE (1-3 cycles) plus 3DCRT/IMRT 4-6 weeks later. Maintenance Sorafenib will be administered only for the patients with non-progression disease 4 to 6 weeks after the completion of radiotherapy. The dose will be 400 mg, p.o., twice a day. Sorafenib will be continuously given for 12 months unless intolerable toxicities and/or tumor progression. If no more than 3 patients discontinue Sorafenib treatment who experience dose-limiting toxicity after necessary dose modification and delay and/or radiation-induced liver disease in the first 15 enrolled patients, the study will recruit second fifteen patients for further evaluating safety and efficacy of treatment. Hypothesis of the current study is that Sorafenib as a maintenance therapy after combined therapy of 3DCRT/IMRT and TACE is safe and superior to radiotherapy combined with TACE alone in terms of time to progression (TTP), progression-free survival (PFS) and overall survival (OS) in comparison to historical data.

**Discussion:**

A recent meta-analysis showed TACE in combination with radiotherapy, improved the survival and the tumor response of patients, and was thus more therapeutically beneficial. In this study, local therapy for HCC is the combination of TACE and radiotherapy. Radiation exposure as a kind of stress might induce the compensatory activations of multiple intracellular signaling pathway mediators, such as PI3K, MAPK, JNK and NF-kB. Vascular endothelial growth factor (VEGF) was identified as one factor that was increased in a time- and dose-dependent manner after sublethal irradiation of HCC cells in vitro, translating to enhanced intratumor angiogenesis in vivo. Therefore, Sorafenib-mediated blockade of the Raf/MAPK and VEGFR pathways might enhance the efficacy of radiation, when Sorafenib is followed sequentially as a maintenance modality. (ClinicalTrials.gov number, NCT00999843.)

## Background

Hepatocellular carcinoma (HCC) is the 6th most common cancer and is the third leading cause of cancer-related death worldwide [1]. In Shanghai, China, HCC was also one of the most common malignancies with the incidence of 41.91/100 000 and 16.52/100 000 for male and female, respectively, which ranked the 4th and 5th according to the epidemiological survey in 2006. Unfortunately, the overall 5-year survival rate for all HCC patients has remained no more than 5% [1]. Surgery is considered as the curative option for HCC. However, when diagnosed, only 20 percent of HCC patients in Shanghai are surgically operable. Whereas, 80% of HCC are either technically unresectable due to local advanced tumor, or medically inoperable due to severe hepatic cirrhosis [2]. For non-surgical managements transcatheter arterial chemoembolization (TACE) has been widely used, and demonstrated its benefit in improving survival of unresectable HCC, compared to the supportive care [3-6], although the benefit was slight.

Three-dimensional conformal radiation therapy (3DCRT)/intensity-modulated radiation therapy (IMRT) has made it possible to escalate irradiation dose to focal HCC without undue dose-limiting toxicity, and has increasingly been recognized as a potentially curative option for patients with HCC [7]. Radiotherapy combined with or without TACE for HCC has shown favorable outcomes in local control and survival with median survival time of 10 months to 25 months [7] and 3-year survival of around 30% [8]. However, intra- and extra-hepatic spreading is still the predominant failure pattern, as reported by Cheng JC et al. They found that after radiotherapy over half of patients failed in intrahepatic multiple recurrences outside irradiation field and extrahepatic metastasis, and only three patients had in-field progression [9]. Liang SX et al reported local failure of 48% and distant metastasis of 19%, including lungs, bones and other sites [8]. In a dose escalation clinical study, which was just completed recently by our institute, out of 40 patients in this trial, 21 patients developed intrahepatic out-of-field failures and 5 had distant metastases (Ren ZG, Zhao JD, Gu K, et al: Three-dimensional conformal radiation therapy and intensity modulated radiation therapy combined with transcatheter arterial chemoembolization for locally advanced hepatocellular carcinoma: an irradiation dose escalation study, submitted). These findings suggest that local and distant failures are still the obstacles for further improvement of outcome in HCC patients after irradiation, and clearly there is a need for novel and more effective therapeutic strategies.

Sorafenib is a multikinase inhibitor with effects against tumor proliferation and angiogenesis. The strong evidence that Sorafenib achieved significantly survival benefit in advanced HCC derived from SHARP trial [10]. In this multi-center randomized study, compared to the placebo arm, patients receiving Sorafenib had a longer median survival (10.7 mo vs 7.9 mo; HR 0.69, P < 0.01) and time to progression (TTP) (HR 0.58, P < 0.01). Another phase III clinical trial concluded that Sorafenib was well tolerated and effective in the treatment of advanced HCC in Asia-Pacific region [11].

The low tumor response rate (less than 5%) with monotherapy of Sorafenib in clinical trials mentioned before indicates that it is unlikely to cure HCC in the absence of local therapies. When Sorafenib is administrated after radiotherapy, it would probably have the effects of preventing or delaying the intrahepatic and extrahepatic spreading of HCC, which provides the rationale for the combination of these treatment modalities.

Hypothesis of the current study is that Sorafenib as a maintenance therapy after combined therapy of 3DCRT/IMRT and TACE is safe, and the outcome is superior to radiotherapy plus TACE in terms of TTP, progression-free survival (PFS) and overall survival (OS) in comparison to historical data.

## Methods and Design

### Study design

**Figure 1:**
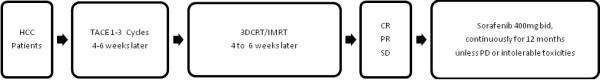
**Treatment Schedule**.

The present study is a non-randomized, uncontrolled single-arm, non-blinded, single-center phase I/II clinical trial. The treatment schedule is shown in Figure 1. Dose-limiting toxicity is defined as follows: Safety and tolerability are evaluated monthly during the period of maintenance Sorafenib. Dose-limiting toxicity (DLT) is defined as: (1) grade 3 hand-foot skin reaction (Table 1); (2) grade 3 and grade 4 non-hematological toxicity, except for hand-foot skin reaction (Table 2); (3) grade 3 and grade 4 hematological toxicity (Table 3); (4) grade 2 to grade 4 hypertension detailed in Table 4. Onset of radiation-induced liver disease (RILD) within 4 months after radiation is an intolerable toxicity, which manifests as either anicteric elevation of alkaline phosphatase (AKP) level of at least two fold of upper normal level (ULN) and nonmalignant ascites (classic RILD) or elevated transaminases of at least five fold of the ULN or of pretreatment level (Grade 3 or 4 hepatic toxicity) (nonclassic RILD). However, progression of HCC should be ruled out for the diagnosis of RILD. Toxicity is graded according to the National Cancer Institute common terminology criteria for adverse events (CTCAE) version 3.0.

**Table 1:** Hand-foot skin reaction grading

Grade 1	Numbness, dysesthesia/paresthesia, tingling, painless swelling or erythema of the hands and/or feet and/or discomfort, which does not disrupt normal activities.
Grade 2	Painful erythema and swelling of the hands and/or feet and/or discomfort affecting the patient's activities.

Grade 3	Moist desquamation, ulceration, blistering or severe pain of the hands and/or feet and/or severe discomfort that causes the patient to be unable to work or perform activities of daily living.

**Table 2:** Non-hematologic criteria for dose delay and dose modification of sorafenib (except for skin toxicity)^#^

Grade	Dose Delay	Dose Modification
Grade 0-2	Treat on time	No Change
Grade 3	Delay* until ≤ Grade 2	Decrease one dose level^
Grade 4		

^#^: Also excludes nausea/vomiting that has not been premedicated, and diarrhea.

*: If no recovery after 30-day delay, treatment will be discontinued.

^: If more than 2 dose reductions are required, treatment will be discontinued.

**Table 3:** Hematologic criteria for dose delay and dose modification of sorafenib

Grade	Dose Delay	Dose Modification
Grade 0-2	Treat on time	No Change
Grade 3	Delay* until ≤ Grade 2	Decrease one dose level^
Grade 4		

*: If no recovery after 30-day delay, treatment will be discontinued.

^: If more than 2 dose reductions are required, treatment will be discontinued.

**Table 4:** Procedure for hypertension emerged by sorafenib treatment

Grade	Management/Next Dose
Grade 1	Consider increased BP monitoring
Grade 2 Asymptomatic and diastolic BP < 110 mm Hg	Begin anti-hypertensive therapy and continue the agent
Grade 2 Symptomatic/persistent or diastolic BP ≥ 110 mmHg or	1. Agent should be held until symptoms resolve and diastolic BP ≤ 100 mmHg; also treat patient with anti-hypertensives and when agent is restarted, reduce by 1 dose level.*
Grade 3	2. If diastolic BP not controlled (≤ 100) on therapy, reduce another dose level.^
Grade 4	Discontinue protocol therapy

*: May be able to resume full dose later.

^: Patients requiring > 2 dose reductions should go off protocol therapy.

If no more than 3 patients discontinue Sorafenib treatment who experience DLT after necessary dose modification which follow the predefined dose levels (Table 5) and delay (Table 2, 3, 4 and 6) and/or RILD in the first 15 enrolled patients, the study will recruit second fifteen patients for further evaluating safety and efficacy of treatment.

**Table 5:** Maintenance sorafenib dose modification table

Dose level 1	Dose level 2	Dose level 3
400 mg b.i.d	400 mg q.d	400 mg q.o.d

200 mg × 2 tablets b.i.d (morning & evening)	200 mg × 2 tablets q.d (morning)	200 mg × 2 tablets q.o.d (morning)

**Table 6:** Skin toxicity criteria for dose delay and dose modification of sorafenib

Toxicity Grade		During a Course of Therapy	Dose for Next Cycle
Grade 1		Maintain dose level^#^	Maintain dose level

Grade 2	1st appearance	Interrupt until resolved to grade 0-1	Maintain dose level
	
	2nd appearance	Interrupt until resolved to grade 0-1	400 mg every day
	
	3rd appearance	Interrupt until resolved to grade 0-1	400 mg every the other day
	
	4th appearance	Discontinue treatment permanently	

Grade 3	1st appearance	Interrupt until resolved to grade 0-1	400 mg every day
	
	2nd appearance	Interrupt until resolved to grade 0-1	400 mg every the other day
	
	3rd appearance	Discontinue treatment permanently	

### Study objectives

The study is designed to investigate the safety, tolerability and also the efficacy of Sorafenib as maintenance for non-progression locally advanced HCC patients, who have received combined therapy of 3DCRT/IMRT and TACE. The primary outcome measure is the safety and tolerability of Sorafenib maintenance for a scheduled period of twelve months. The secondary outcome measures TTP, PFS and OS.

### Trial organization

This trial has been designed by the Department of Radiation Oncology and Integrative Oncology in cooperation with Department of Interventional Radiology. The trial is an investigator-initiated trial and is supported partly by Bayer Schering Pharma, China.

### Coordination and monitoring

The trial is coordinated by the clinical trial office of Fudan University Shanghai Cancer Center, Shanghai, China. During patient recruitment in this center monitoring on site is performed according to good clinical practice (GCP) guidelines. The trial is registered with the ClinicalTrials.gov (NCT00999843). The data management will be performed by the clinical trial office of Fudan University Shanghai Cancer Center, Shanghai, China.

### Ethical approval

The final protocol was approved on February 2, 2009 by Research Ethics Committee of Fudan University Shanghai Cancer Center (Reference number: 090168-9).

### Patient selection

All of inclusion criteria should be satisfied:

1. Age of equal or older than 18 years with a life expectancy of at least 12 weeks;

2. Karnofsky performance status (KPS) of ≥70;

3. Histologically or cytologically confirmed HCC;

4. BCLC stage B, solitary lesion (bigger than 5 cm in diameter) with tumor burden less than 50% of total liver volume;

5. Liver function of Child-Pugh A;

6. Technically unresectable, medically inoperable, or surgery declined by the patient;

7. Adequate renal function (serum creatinine concentration ≤ 1.5 ×ULN), and hepatic function (serum total bilirubin level ≤ 2 × ULN, serum aspartate and alanine transaminase levels ≤ 2.5 × ULN); and adequate bone marrow reservation (absolute neutrophil count ≥ 1500 cells/mm^3^, platelet count ≥ 60 000 cells/mm^3^, and hemoglobin ≥ 8.5 g/dL); and prothrombin time ≤ 1.5 × ULN;

8. Signed informed consent must be obtained prior to any study specific procedure.

The followings are the exclusion criteria:

1. Presence of intrahepatic and/or extrahepatic metastases

2. Previous received systemic therapy for liver cancer;

3. History of radiotherapy to the liver;

4. Indistinct tumor boundary on CT/MRI images;

5. Previous or concurrent malignancies, with the exception of adequately treated basal cell carcinoma of the skin or in situ carcinoma of the cervix or superficial bladder tumors [T_a_, T_is _and T_1_];

6. History of cardiac disease: congestive heart failure > NYHA class 2, active CAD, cardiac arrythmias requiring anti-arrhythmic therapy or uncontrolled hypertension within the last 12 months;

7. Concurrent uncontrolled medical conditions and recent bleeding diathesis;

8. Pregnancy or breast feeding;

9. Investigational drug therapy outside of this trial during or within 4 weeks of study entry;

10. Psychiatric or medical unstable conditions that compromise the patient's ability to give informed consent.

### TACE before radiation therapy

TACE as a standard treatment for this specific patient population will be implemented before radiotherapy. The chemotherapeutic agents injected are a combination of one platinum-containing drug, for instance, cis-platinum (DDP), carbon platinum (CBP) or Oxaliplatin (L-OHP) and one anti-tumor antibiotics drug, for instance, mitomycin (MMC), adrimycin (ADM) or Epi-ADM. TACE will be carried out with an interval of 4-6 weeks between cycles. Total course of TACE will be terminated if more than 75% of tumor volume occupied by iodine oil in CT scan one month after 1, 2 or 3 cycles of TACE. From our previous experience, if three cycles of TACE have not achieved adequate iodine deposition, the chance of getting more iodine accumulation is quite low with furthermore TACE. Moreover, excessive TACE would cause more severe damage to Chinese HCC patients with hepatic cirrhosis. Therefore, at most 3 cycles of TACE will be carried out before radiation therapy.

### Radiation therapy

Radiotherapy is delivered 4-6 weeks after the last cycle of TACE. Active breath coordinator (ABC) is applied to control the liver motion due to respiration [12]. Patients undergo CT simulation with individualized immobilization vacuum lock. Helical CT scan is performed with 5 mm of thickness and a pitch of 1:1. Scan scope is from 3-4 cm above the dome of diaphragm to the level below right kidney. Gross tumor volume (GTV) in liver is delineated on the axial simulation CT images with the aid of deposited iodinated-oil, MRI, or contrast enhanced CT images. Clinical target volume (CTV) is formed by adding a uniform 5-8 mm around GTV within the liver. The extra margin needed for internal target volume (ITV) is individually decided based on the reproducibility of lesion/liver position when ABC is applied. Planning target volume (PTV) is formed by adding a margin of 6 mm around ITV. 3DCRT treatment plan is preferred, but IMRT would also be considered when dose constrains of normal organs and tumor could not be satisfied with 3DCRT. For 3DCRT or IMRT plan, multiple co-planar or non-coplanar portals are designed with the intention to reduce the dose to normal liver and the normal liver volume irradiated as much as possible. For IMRT plan, we use a simplified IMRT technique, which applies: (1). The number of portals is ≤ 5; (2). The number of beam lit (subfield) is ≤ 5; (3). The size of the smallest subfield is ≥ 5 cm × 5 cm. The radiation dose prescribed to tumor is as high as possible, but the maximum doses are 62 Gy and 52 Gy for patients with tumor diameter less than 10 cm and ≥ 10 cm, respectively. The dose is delivered by conventional fractionation, i.e., 2 Gy/fraction, one fraction per day and 5 fractions per week.

The organs at risk (OAR) contoured include liver, bilateral kidneys, spinal cord, stomach and duodenum. Dose constraint to liver is mean dose to normal liver (MDTNL) (total liver volume minus PTV) of ≤ 23 Gy, and the dose volume histogram (DVH) is in a tolerable area based on our previous experience: V_5 _of <86%, V_10 _of <68%, V_15 _of <59%, V_20 _of <49%, V_25 _of <35%, V_30 _of <28%, V_35 _of <25%, and V_40 _of <20% [13]. Should one kidney receive >20 Gy, >90% of the other kidney should be excluded from the primary beam. Maximal permitted dose to spinal cord is 45 Gy. Maximal permitted dose to stomach and duodenum is 50 Gy for any volume of 1 mm^3^.

### Maintenance of Sorafenib

Sorafenib is administered only to the patients with non-progression disease (CR, PR and SD) 4 weeks after the completion of radiotherapy. The dose is 400 mg, p.o., twice a day. Sorafenib is continuously given for 12 months unless intolerable toxicities and/or tumor progression. Doses would be delayed or reduced for clinically significant hematologic, or other toxicities that are related to Sorafenib. If a patient experiences several toxicities and there are conflicting recommendations, the recommended dose adjustment that reduces the dose to the lowest level would be used. All dose modifications follow the predefined dose levels (Table 5). A grading scale is used for hand-foot skin reaction (HFS) (Table 1), and specific dose modifications are implemented (Table 6). Non-hematologic and hematologic criteria for dose delay and dose modification of Sorafenib are shown in Table 2 and 3. Table 4 shows the management procedure of hypertension resulted from Sorafenib. Patients are followed up monthly during Sorafenib adminstration.

### Evaluation and follow-up

Treatment-related toxicity is assessed according to CTCAE version 3.0, and is evaluated weekly during TACE and irradiation, monthly for the period of Sorafenib and every half-year thereafter. Each visit includes physical examination, complete blood count and blood chemistry.

Abdominal CT/MRI, chest CT, bone scan and serum AFP are performed right after completion of TACE and irradiation, and repeated every two months.

Sorafenib-related toxicities will be assessed during Sorafenib administration until tumor progression. Once intrahepatic or extrahepatic failures are suspected, the corresponding examinations would be carried out.

### Statistics

Tumor response is assessed using Response Evaluation and Criteria in Solid Tumors (RECIST) during every visit. Tumor progression is defined as one of the followings: (1). Hepatic tumor enlarges in size after it shrinks or remains stable; (2). New intrahepatic lesion occurs; (3). Extrahepatic nodes or distant metastasis develop. The enhanced AFP level in serum after declined without image or clinical evidences are not considered to be tumor progression. The time from the commencement of TACE to tumor progression is counted to get TTP. The other endpoints are PFS and OS. All of the rates are estimated by Kaplan-Meier method, and all observations start on the date of the first TACE. The sample size is calculated as follows: The null hypothesis of a 2-year local progression-free rate of about 40% is based on the result of a radiation dose escalation clinical trial recently completed in our department with the same TACE and radiotherapy techniques, in which the study accumulated 40 patients (Ren ZG, Zhao JD, Gu K, et al: Three-dimensional conformal radiation therapy and intensity modulated radiation therapy combined with transcatheter arterial chemoembolization for locally advanced hepatocellular carcinoma: an irradiation dose escalation study, submitted). This is tested against the alternative hypothesis of a true rate of 70% or higher with an α level of 5% and a power of 80%, which required 24 patients according to the method proposed by Makuch and Simon [14]. Taking the drop-out rate of 20% into consideration, a total of 30 patients will be recruited. Accrual will be completed in 12 months. A median follow-up period will be 30 months.

## Discussion

In this study, local therapy is the combination of TACE and radiotherapy. Necrosis after TACE is usually incomplete because of large tumor volume [15,16], thus 70%-80% of patients finally succumb to intrahepatic tumor progression [3]. A recent meta-analysis showed that TACE in combination with radiotherapy, compared to TACE alone, significantly improved the survival and the tumor response, and was thus more therapeutically beneficial [17]. Furthermore, implement of TACE before radiotherapy has the additional advantages of deposited iodine oil for target contouring and verification.

Sorafenib inhibits the activity of targets present in the tumor cell, including members of the Raf family of serine/threonine kinases. In addition, Sorafenib inhibits receptor tyrosine kinases, including Flt-3, kit, Ret, vascular endothelial growth factor receptor-2 (VEGFR-2), vascular endothelial growth factor receptor-3 (VEGFR-3), and platelet-derived growth factor receptor beta (PDGFR-beta) [18,19]. Radiation exposure as a kind of stress might induce the compensatory activations of multiple intracellular signaling pathway mediators, such as PI3K, MAPK, JNK and NF-kB [20]. VEGF was identified as one factor that was increased in a time- and dose-dependent manner after sublethal irradiation damage of HCC cells in vitro, translating to enhanced intratumor angiogenesis in vivo and correlating well with serum VEGF levels [21]. Therefore, Sorafenib-mediated blockage of the Raf/MAPK and VEGFR pathways might enhance the efficacy of radiation, when Sorafenib is followed sequentially. In HCT116 xenograft tumor growth delay experiments in mice, Sorafenib altered radiation response in a schedule-dependent manner. Radiation followed sequentially by Sorafenib was found to be associated with the greatest tumor growth delay [22].

## Competing interests

The authors declare that they have no competing interests.

## Authors' contributions

JDZ and GLJ developed the study and wrote the initial study protocol. ZYX, WTL, LML, ZC, ZHZ and GLJ are study coordinators. JDZ, JL, ZGR and KG wrote the manuscript and GLJ supported the development of the study protocol. All authors read and approved the final manuscript.
